# Eucalyptus Wood Smoke Extract Elicits a Dose-Dependent Effect in Brain Endothelial Cells

**DOI:** 10.3390/ijms251910288

**Published:** 2024-09-24

**Authors:** Dorothy J. You, Bria M. Gorman, Noah Goshi, Nicholas R. Hum, Aimy Sebastian, Yong Ho Kim, Heather A. Enright, Bruce A. Buchholz

**Affiliations:** 1 Biosciences and Biotechnology Division, Physical and Life Sciences Directorate, Lawrence Livermore National Laboratory, Livermore, CA 94550, USA; you5@llnl.gov (D.J.Y.);; 2 Public Health and Integrated Toxicology Division, Center for Public Health and Environmental Assessment, U.S. Environmental Protection Agency, Research Triangle Park, NC 27709, USA; 3 Center for Accelerator Mass Spectrometry, Lawrence Livermore National Laboratory, Livermore, CA 94550, USA

**Keywords:** wildfire smoke, wood smoke, climate change, brain endothelial cells, HBMEC, hCMEC/D3, nuclear factor erythroid 2-related factor 2 (NRF2), aryl hydrocarbon receptor (AhR)

## Abstract

The frequency, duration, and size of wildfires have been increasing, and the inhalation of wildfire smoke particles poses a significant risk to human health. Epidemiological studies have shown that wildfire smoke exposure is positively associated with cognitive and neurological dysfunctions. However, there is a significant gap in knowledge on how wildfire smoke exposure can affect the blood–brain barrier and cause molecular and cellular changes in the brain. Our study aims to determine the acute effect of smoldering eucalyptus wood smoke extract (WSE) on brain endothelial cells for potential neurotoxicity in vitro. Primary human brain microvascular endothelial cells (HBMEC) and immortalized human brain endothelial cell line (hCMEC/D3) were treated with different doses of WSE for 24 h. WSE treatment resulted in a dose-dependent increase in IL-8 in both HBMEC and hCMEC/D3. RNA-seq analyses showed a dose-dependent upregulation of genes involved in aryl hydrocarbon receptor (AhR) and nuclear factor erythroid 2-related factor 2 (NRF2) pathways and a decrease in tight junction markers in both HBMEC and hCMEC/D3. When comparing untreated controls, RNA-seq analyses showed that HBMEC have a higher expression of tight junction markers compared to hCMEC/D3. In summary, our study found that 24 h WSE treatment increases IL-8 production dose-dependently and decreases tight junction markers in both HBMEC and hCMEC/D3 that may be mediated through the AhR and NRF2 pathways, and HBMEC could be a better in vitro model for studying the effect of wood smoke extract or particles on brain endothelial cells.

## 1. Introduction

The total number and intensity of wildfires have been increasing globally in recent decades [1,2]. Particulate matter 2.5 (PM_2.5_), fine particles less than 2.5 μm, from wildfire smoke is a significant source of air pollution and poses a significant health hazard [3,4,5]. During the Canadian wildfire season in 2023, the peak wildfire PM_2.5_ level reached 317 μg/m^3^ in New Jersey, which is ten times higher than the 24 h average limit of the National Ambient Air Quality Standard [6]. It is well established that exposure to wildfire PM_2.5_ is associated with an increased risk for respiratory and cardiovascular diseases [7,8]. However, a recent study has shown that wildfire smoke exposure not only affects respiratory and cardiovascular systems but also affects cognitive and neurological functions [9]. Epidemiological data suggest that a high level of wood smoke exposure can be associated with an increased risk for Alzheimer’s disease, Parkinson’s disease, and dementia [10,11,12]. In addition, individuals who were exposed to the 2018 Camp Fire in California showed significant cognitive deficits, especially in interference processing and frontal brain function, when tested 6–12 months after the fire exposure [13]. More recently, wildfire smoke exposure has been shown to result in neuroinflammation, including proinflammatory phenotypes of microglia and peripheral immune cells, in the brains of normal C57BL/6 mice [14].

Inhaled particulate matter from different sources has been shown to enter the bloodstream or traverse the olfactory nerve directly to reach the brain [15,16]. Particulate matter from tobacco smoke and traffic-related air pollution have been shown to cause inflammatory responses in the brain and impairment of blood–brain barrier (BBB) homeostasis [17,18,19,20,21]. Inhaled vehicle traffic air pollution particles can be deposited in the hippocampal region of rats, whereas cigarette smoke can induce neuroinflammation that could be a secondary response to oxidative stress [20,22]. Furthermore, PM_2.5_-polluted human brain models created using a 3D microfluidic platform showed that PM_2.5_ can penetrate across the BBB and accumulate in brain tissue to cause inflammation [23]. The BBB is the gatekeeper of the brain, shielding the brain from toxic substances within the bloodstream while supplying the brain tissue with nutrients [24,25]. At the same time, the BBB also filters harmful compounds from the brain back into the bloodstream for metabolism and excretion [24,25]. Inflammation in the brain results in an increase in ICAM-1, VCAM-1, and selectin to recruit and control the adherence of monocytes and neutrophils to vessel walls affecting the integrity of the BBB [26,27,28,29]. BBB endothelial cells demonstrate a significant barrier function and are characterized by the presence of complex tight junctions, including occludin, claudins, and zonula occludens-1, -2 (ZO-1 and ZO-2), and the dysregulation of these proteins has been associated with altered BBB permeability [30]. Animal studies have shown that gasoline and diesel exhaust exposure resulted in dysregulation of tight junction protein expression including claudin-5 and occludin [31]. Similar studies observed that nicotine disturbed tight junction proteins, including claudin-1, -3, -5, and ZO-1 and ZO-2, and increased BBB permeability [32,33]. Thus, brain endothelial cells have been widely used as an in vitro model to identify specific mechanisms of neuroinflammation in the brain, and exposure to particulate matter from cigarette smoke, diesel exhaust, and fine dust particles resulted in inflammatory responses that disrupted the function, integrity, and permeability of the cells [23,32,34,35,36,37,38,39,40]. While studies have shown that exposure to different sources of particulates can elicit molecular and cellular changes in brain endothelial cells in vitro, no studies yet have shown how exposure to wildfire smoke or wood smoke can elicit cellular inflammatory responses in brain endothelial cells in vitro [34,35,36,37,38,39,40].

A complex mixture of chemicals in wildfire smoke hinders the interpretation of the exact mechanisms of toxicity compared to the other particulates, especially when using in vivo models [41,42]. Chemical analysis of the wildfire smoke from the 2018 Camp Fire in California showed varying levels of metals, organic carbon compounds, and phosphorous flame retardants that could cause DNA damage [43,44,45]. Furthermore, different stages of combustion, including flaming and smoldering phases, result in different compositions of smoke particles [46]. The smoldering phase of fire produces a larger amount of particulate matter and lasts longer, which can result in chronic exposure to smoke [46,47]. Moreover, a previous study has shown that smoldering eucalyptus wood smoke extract (WSE) resulted in the highest lung toxicity compared to other types of biomass smoke, including red oak and pine woods [46]. Thus, we decided to take an in vitro approach to determine the effect of WSE on brain endothelial cells for potential neurotoxicity.

The goal of this study is to investigate the acute effect of WSE in brain endothelial cells using both primary human brain microvascular endothelial cells (HBMEC) and immortalized human brain endothelial cell line (hCMEC/D3). Based on previous studies, we hypothesize that WSE will induce proinflammatory cytokine production and disturb the tight junctions in both HBMEC and hCMEC/D3. From our study, we found that both HBMEC and hCMEC/D3 treated with WSE for 24 h induce genes involved in the aryl hydrocarbon receptor (AhR) and nuclear factor erythroid 2-related factor 2 (NRF2) pathways to elicit IL-8 production dose-dependently.

## 2. Results

### 2.1. Dose-Dependent Increase in IL-8 Proinflammatory Cytokine Production in HBMEC and hCMEC/D3 Treated with WSE

**Figure 1:**
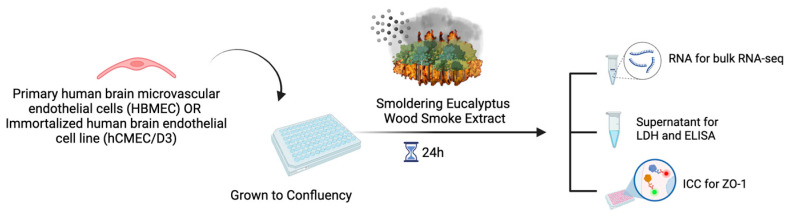
Experimental design for this study.

HBMEC were treated with either 10, 30, or 50 µg/mL or 20, 70, or 100 µg/mL of WSE for 24 h (Figure 1).

**Figure 2:**
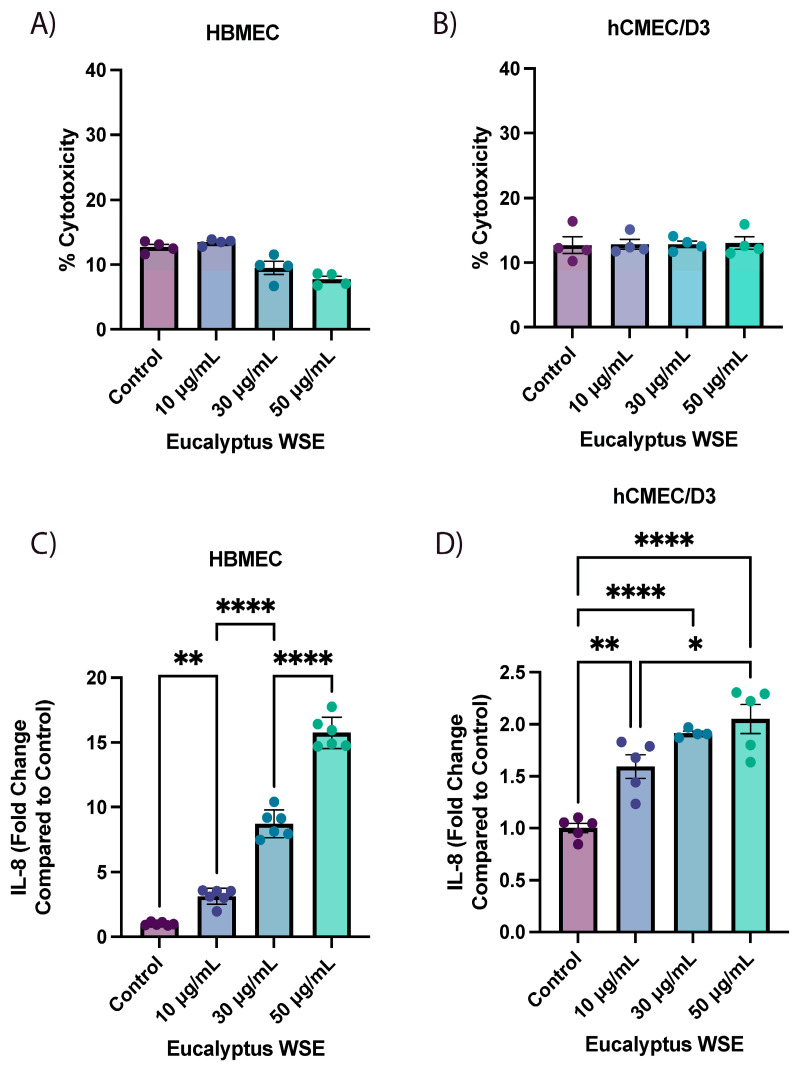
Human brain microvascular endothelial cells (HBMEC) and immortalized human brain endothelial cell line (hCMEC/D3) treated with 10, 30, or 50 µg/mL of smoldering eucalyptus wood smoke extract (WSE) for 24 h. Lactate dehydrogenase (LDH) activity measured from cell supernatants in (**A**) HBMEC and (**B**) hCMEC/D3 (n = 4/treatment group). Secreted IL-8 protein levels were measured from cell supernatants by an enzyme-linked immunosorbent assay (ELISA) in (**C**) HBMEC and (**D**) hCMEC/D3 (n = 4–6/treatment group, **** *p* < 0.0001, ** *p* < 0.01, and * *p* < 0.05 compared to listed treatments using one-way ANOVA with post-Tukey’s test).

The cytotoxicity of each treatment was measured by a lactate dehydrogenase (LDH) assay as described in the Materials and Methods Section. We found no significant cytotoxicity with 10, 20, 30, 50, or 70 µg/mL WSE, whereas 100 µg/mL WSE was significantly cytotoxic (Figure 2A and Supplementary Figure S1). Supernatants from HBMEC treated with WSE (20, 70, 100 µg/mL) were used in XL Proteome Profiler to identify and determine cytokine production. Only 1 proinflammatory cytokine out of 105 cytokines, IL-8, was significantly increased in 20 and 70 µg/mL of WSE compared to the control in XL Proteome Profiler. With a significant increase in cytotoxicity from treatment with 100 µg/mL of WSE in HBMEC, hCMEC/D3 was treated with 10, 30, or 50 µg/mL WSE for 24 h, and no significant cytotoxicity was found in the LDH assay (Figure 2B). The secreted IL-8 protein level was increased dose-dependently in HBMEC and hCMEC/D3 that were treated with 10, 30, or 50 µg/mL of WSE (Figure 2C,D). The dose-dependent effect on IL-8 production was much more significant compared to control and was much greater in HBMEC compared to hCMEC/D3 (Figure 2C,D).

### 2.2. Dose-Dependent Induction of Genes Related to AhR and NRF2 Pathways in HBMEC and hCMEC/D3 Treated with WSE

**Figure 3:**
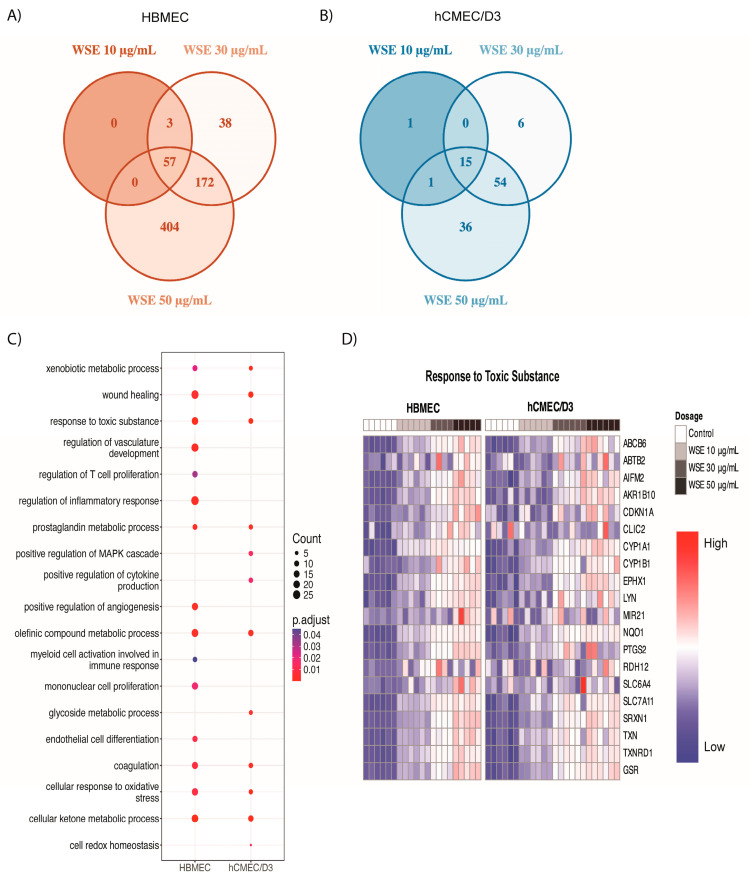
Bulk RNA sequencing (RNA-seq) analyses of upregulated differentially expressed genes (DEGs). Venn diagram illustrating the overlap and unique upregulated DEGs between WSE treatments (10, 30, 50 µg/mL). The numbers indicate the upregulated DEGs in each treatment group for (**A**) HBMEC and (**B**) hCMEC/D3, highlighting both shared and distinct gene expression patterns. Gene Ontology (GO) enrichment analyses were performed with upregulated DEGs in HBMEC and hCMEC/D3. (**C**) Significant and relevant biological process in HBMEC and hCMEC/D3. (**D**) Heatmap of genes showing the upregulated genes in both HBMEC and hCMEC/D3 treated with WSE for the biological process involved in response to toxic substance.

To better understand WSE-induced changes at the molecular level, we profiled transcriptional changes in both HBMEC and hCMEC/D3 treated with 10, 30, or 50 µg/mL WSE for 24 h using bulk RNA sequencing (RNA-seq). During the identification of differentially expressed genes (DEGs), we found that 57 genes were significantly upregulated in all doses of WSE treatment in HBMEC and a number of significantly increased genes were dose-dependently increased in HBMEC (Figure 3A). Only 15 genes were significantly upregulated in all doses of WSE treatment in hCMEC/D3, but a number of significantly upregulated genes also increased dose-dependently for hCMEC/D3 (Figure 3B). Gene Ontology (GO) enrichment analyses were performed to determine the functions of DEGs in both HBMEC and hCMEC/D3 between the control and 50 µg/mL WSE groups. The GO enrichment analysis showed that genes upregulated in response to WSE were associated with similar biological processes for both HBMEC and hCMEC/D3, including xenobiotic metabolic processes, wound healing, responses to toxic substances, and cellular responses to oxidative stress (Figure 3C). Mononuclear cell proliferation, myeloid cell activation involved in immune response, endothelial cell differentiation, and regulation of T cell proliferation were only increased in HBMEC, while positive regulation of the mitogen-activated protein kinases cascade, glycoside metabolic process, and positive regulation of cytokine production were increased in hCMEC/D3 only. Many genes involved in response to toxic substances, including *CYP1A1*, *CYP1B1*, *GSR*, *NQO1*, and *TXNRD1*, increased dose-dependently in both HBMEC and hCMEC/D3 (Figure 3D).

**Figure 4:**
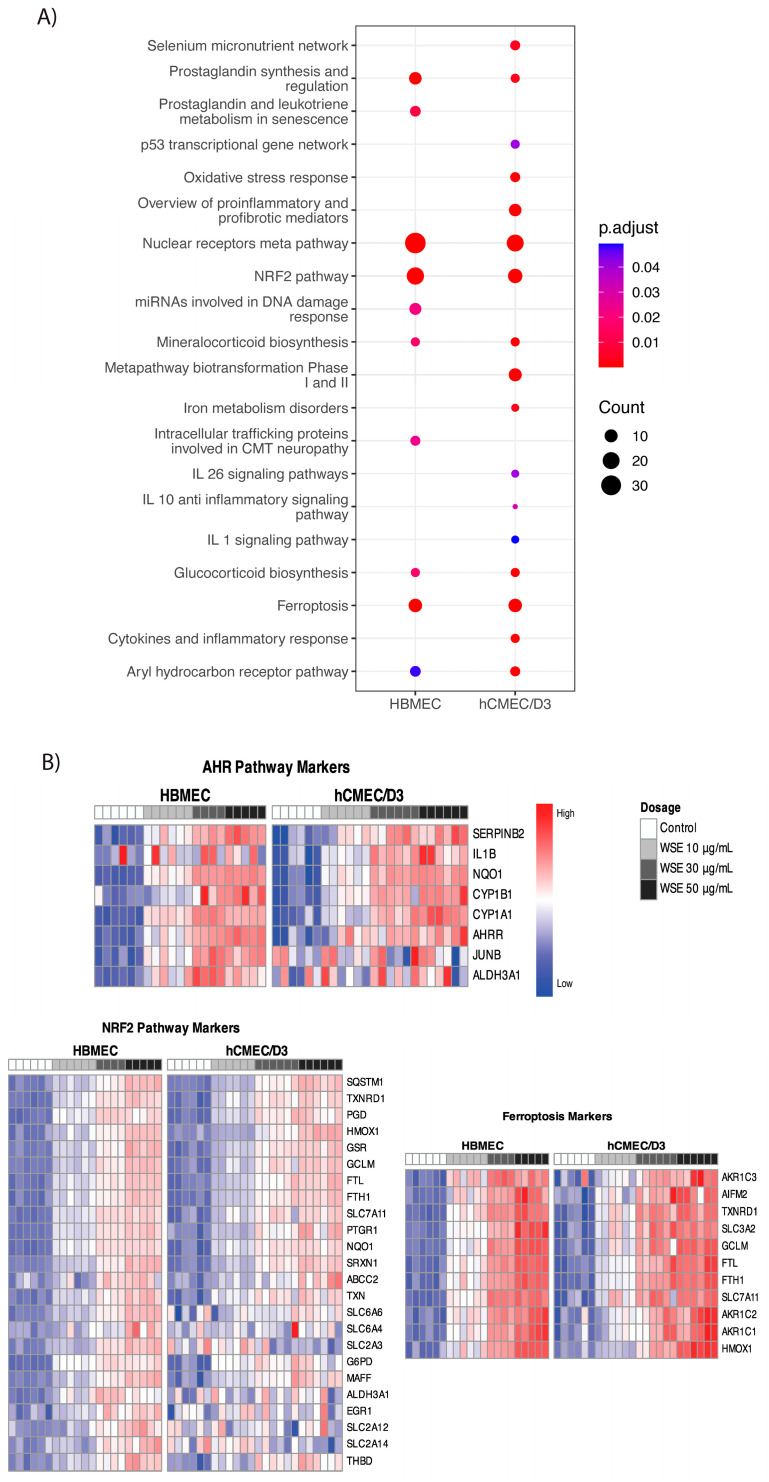
Differential gene expression in HBMEC and hCMEC/D3 treated with different levels of WSE (0, 10, 30, 50 µg/mL) for 24 h. (**A**) Dot plots showing enriched pathways using the WikiPathway database. (**B**) Heatmap of genes showing the upregulated genes in both HBMEC and hCMEC/D3 for pathways including the AhR pathway, NRF2 pathway, and ferroptosis.

Pathway analysis is critical for determining the potential signaling mechanisms involved in the induction of inflammatory cytokine production in these cells following WSE treatment. Pathway analysis for DEGs between the control and 50 µg/mL WSE groups using the WikiPathway database showed that similar pathways were significantly increased in both HBMEC and hCMEC/D3, including the NRF2 pathway, aryl hydrocarbon receptor pathway, nuclear receptor meta pathway, and ferroptosis (Figure 4A). The selenium micronutrient network, p53 transcription gene network, oxidative stress repones, iron metabolism disorders, and interleukin signaling pathways were increased in hCMEC/D3 only. Similar changes were observed in HBMEC and hCMEC/D3, showing a dose-dependent increase in transcripts that are involved in AhR pathway genes (e.g., *IL1B*, *NQO1*, *CYP1B1*, *CYP1A1*, and *AHRR*), NRF2 pathway genes (e.g., *HMOX1*, *GSR*, *GCLM*, *FTL*, *FTH1*, and *NQO1*), and ferroptosis genes (e.g., *GCLM*, *FTL*, and *FTH1*) (Figure 4B). Furthermore, other genes that significantly increased in HBMEC included *MIR222*, which has been indicated in brain injury, and *NPTX1*, *MIR27A*, and *MMP1*, genes shown to be upregulated when exposed to different types of smoke (Supplementary Figure S2) [48,49,50,51]. Although many genes and pathways related to reactive oxygen species (ROS) production and metabolism were increased, no significant changes in ROS production were observed in HBMEC and hCMEC/D3 treated with different levels of WSE for 24 h from the DCFDA assay (Supplementary Figure S3).

### 2.3. Significantly Different Gene Ontology Terms Were Detected in Downregulated Genes between HBMEC and hCMEC/D3 Treated with WSE

**Figure 5:**
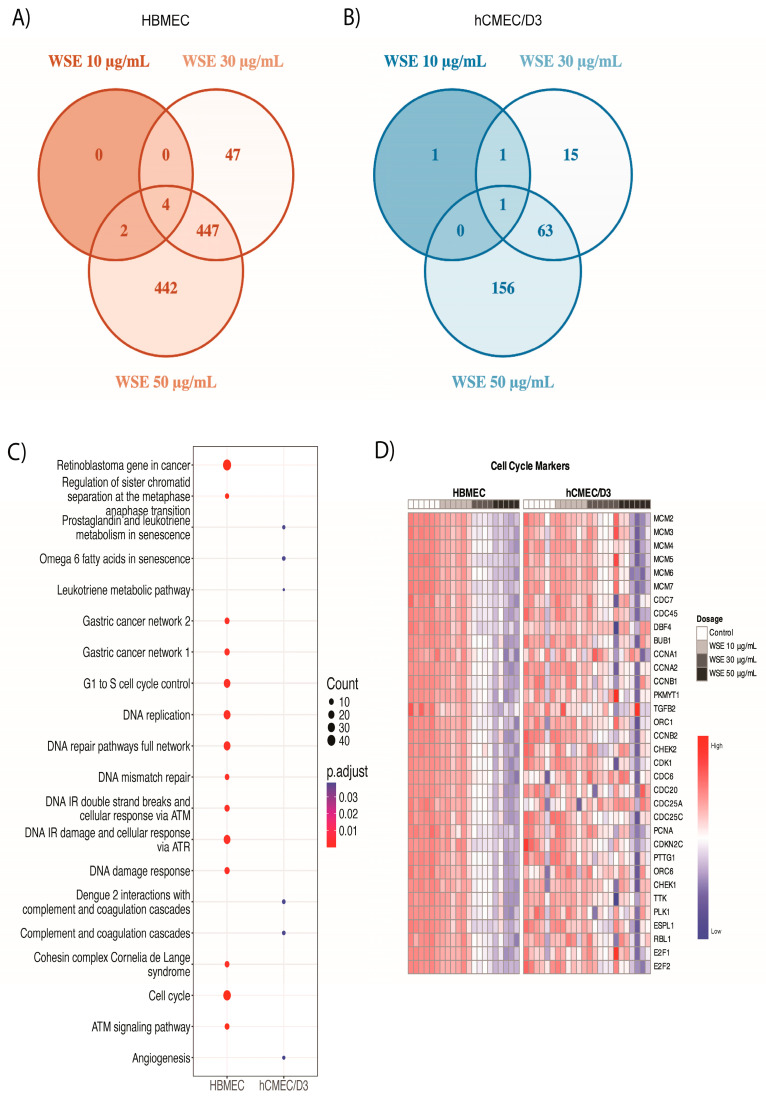
RNA-seq analyses of downregulated DEGs. Venn diagram of downregulated DEGs between WSE treatments (10, 30, 50 µg/mL). The numbers indicate downregulated DEGs in each treatment group for (**A**) HBMEC and (**B**) hCMEC/D3. Gene Ontology enrichment analyses were performed with downregulated DEGs in HBMEC and hCMEC/D3. (**C**) Dot plots showing enriched pathways using WikiPathway. (**D**) Heatmaps of cell cycle markers.

We then compared downregulated genes between HBMEC and hCMEC/D3 treated with 10, 30, or 50 µg/mL WSE for 24 h. We found that 895 genes were significantly downregulated in 50 µg/mL WSE during the identification of DEGs, while only 4 of these genes were downregulated in all doses of WSE in HBMEC (Figure 5A). For hCMEC/D3, 220 genes were significantly downregulated in 50 µg/mL WSE while 1 gene was downregulated in all doses of WSE in hCMEC/D3 (Figure 5B). For both HBMEC and hCMEC/D3, all downregulated changes were dose-dependent. GO enrichment analyses between control and 50 µg/mL WSE showed that distinct terms were downregulated in HBMEC and hCMEC/D3, unlike what we observed in the upregulated genes. For cellular components, HBMEC resulted in a significant decrease in genes involved in DNA replication, spindle pole, replication fork, microtubule, condensed chromosome, and the chromosomal region, whereas hCMEC/D3 resulted in a decrease in genes involved in the external side of the plasma membrane and collagen trimmer (Supplementary Figure S4A). Genes involved in the collagen-containing extracellular matrix were downregulated in both HBMEC and hCMEC/D3 (Supplementary Figure S4A). When comparing biological processes, genes involved in cell cycle checkpoint signaling, the regulation of cell cycle phase transition, organelle fission, and DNA replication were decreased in HBMEC, whereas hCMEC/D3 showed a decrease in genes involved in leukocyte migration, the regulation of innate immune response, chemokine production, and humoral immune response for biological processes (Supplementary Figure S4B). No biological processes overlapped between HBMEC and hCMEC/D3 for downregulated genes. Furthermore, pathway analysis showed a significant decrease in the cell cycle and many of the genes related to the cell cycle (e.g., DNA replication and G1-to-S cell cycle control) in HBMEC, whereas hCMEC/D3 exhibited a decrease in the leukotriene metabolic pathway, angiogenesis, and complement and coagulation cascade (Figure 5C). Most of the cell cycle markers were consistently decreased dose-dependently in HBMEC while no significant changes were observed in hCMEC/D3 (Figure 5D). Some of the genes related to cell cycle arrest and senescence were examined, including *CDKN1A*, *CDKN2A*, *CKDN2B*, *E2F1*, *E2F2*, and *DHFR* (Supplementary Figure S5) [52,53,54,55,56,57]. Senescence is a stable cell cycle arrest and hallmark of aging, but different stimuli or cellular stressors can induce senescence [58]. An increase in *CDKN1A* and *CDKN2B* and a decrease in *DHFR, E2F1,* and *E2F2* were significant between the control and 50 µg/mL WSE treatment groups in HBMEC only (Supplementary Figure S5).

### 2.4. HBMEC Express Increased Levels of Tight Junction Markers Compared to hCMEC/D3

**Figure 6:**
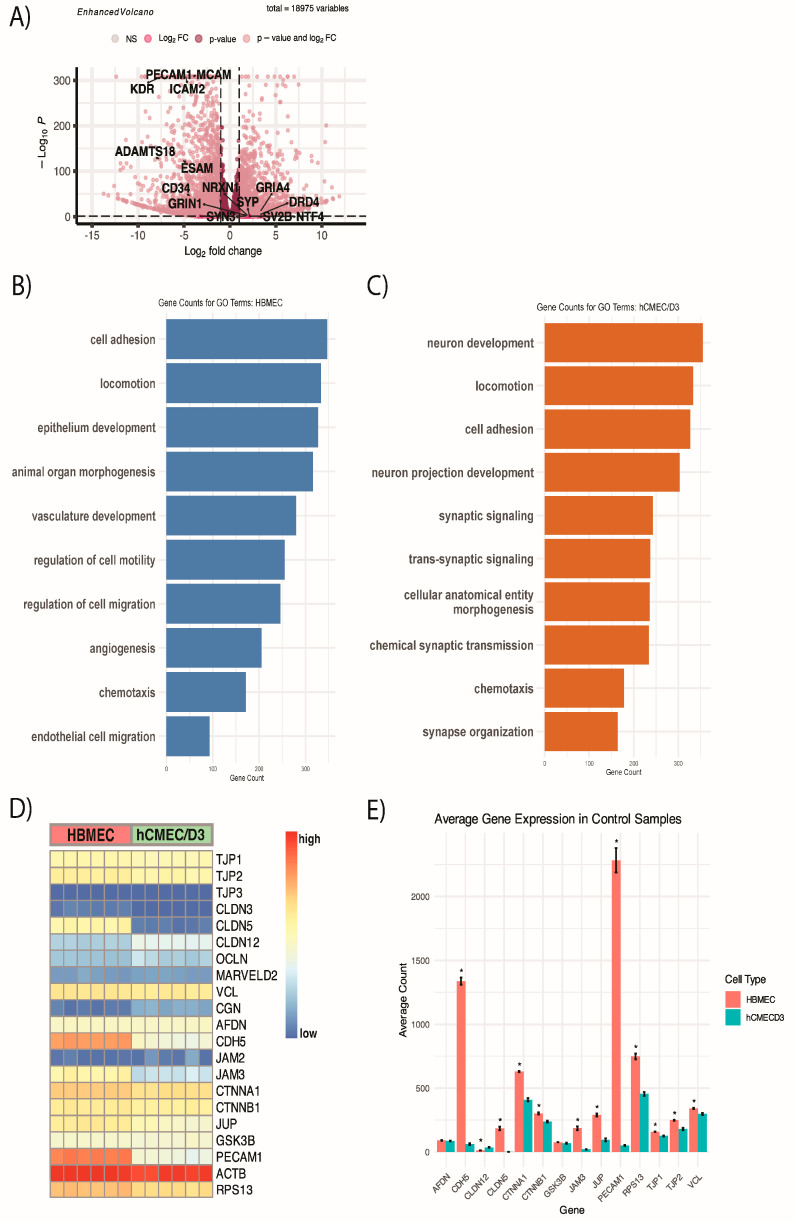
Comparison of untreated control transcript expression levels between HBMEC and hCMEC/D3. (**A**) Volcano plot for both upregulated and downregulated DEGs from the comparison between controls of HBMEC and hCMEC/D3. Significant and relevant biological processes in (**B**) HBMEC and (**C**) hCMEC/D3. (**D**) Heatmap of tight junction markers in HBMEC and hCMEC/D3. (**E**) Average count of endothelial cell and tight junction markers in HBMEC and hCMEC/D3. (* *p* < 0.05 between two cell types using Student’s *t*-test).

To better understand the differences observed in the two endothelial cell types we used in the study, we compared untreated controls between HBMEC and hCMEC/D3 using RNA-seq analyses. The volcano plot shows that primary HBMEC express significantly higher endothelial cell markers, including *PECAM-1*, known to play an important role in the tight junctions of endothelial cells, whereas hCMEC/D3 expresses genes related to synapse formation such as *NRXN1*, *DRD4*, and *NTF4* (Figure 6A) [59]. These patterns were also observed when GO enrichment analyses were performed between untreated HBMEC and hCMEC/D3 controls. Gene counts were significantly higher for epithelium development, vasculature development, and angiogenesis for HBMEC, whereas neuron development, neuron projection development, and synaptic signaling were higher in hCMEC/D3 (Figure 6B,C). Both had increased locomotion, cell adhesion, and chemotaxis. Although there were some differences in biological processes between HBMEC and hCMEC/D3, both cells exhibited similar endothelial cell and tight junction markers, including *ZO-1,2 (TJP1,2), CLDN-5*, *VCL*, *AFDN*, *CTNNA-A1*, *B1*, and *RPS13* (Figure 6D,E) [60]. However, HBMEC had higher expression of most endothelial cell and tight junction markers, including *CLDN-5*, *PECAM-1*, *ZO-1*, and *ZO-2* (*TJP1* and *TJP2*) compared to hCMEC/D3.

### 2.5. Changes in Tight Junction and Gap Junction Markers in HBMEC and hCMEC/D3 Treated with WSE

**Figure 7:**
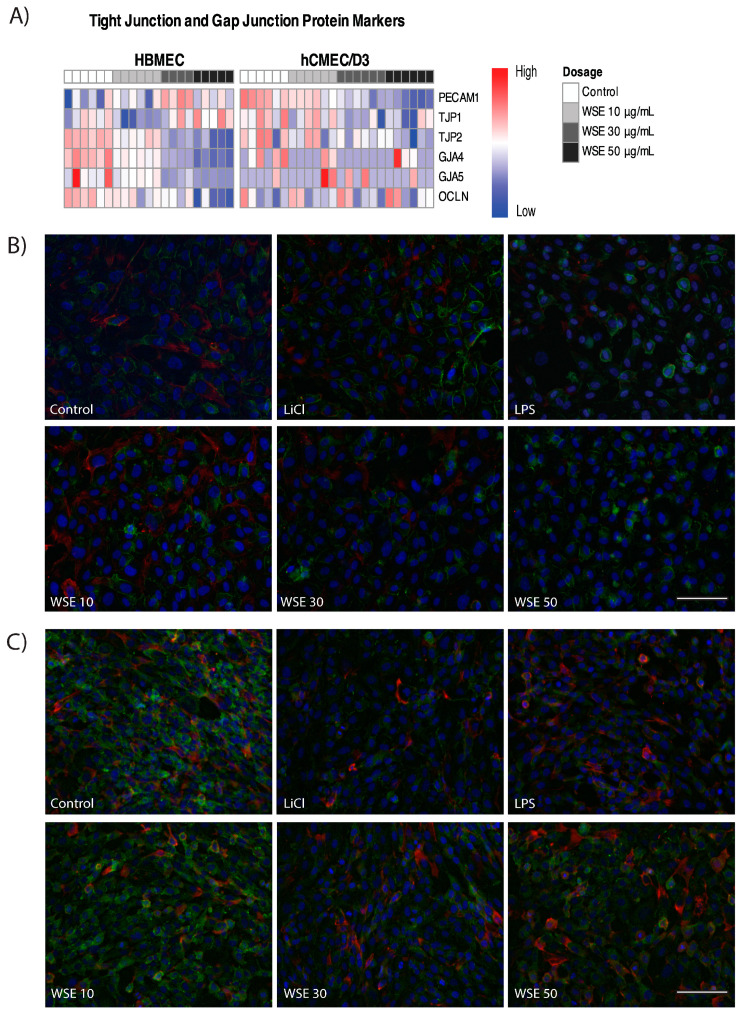
Changes in tight junction markers in HBMEC and hCMEC/D3 treated with WSE for 24 h. (**A**) Key differentially expressed genes in HBMEC and hCMEC/D3 treated with 10, 30, or 50 µg/mL of WSE. Representative images of ZO-1 staining for (**B**) HBMEC and (**C**) hCMEC/D3 treated with 10, 30, or 50 µg/mL of WSE, lipopolysaccharide (LPS), or lithium chloride (LiCl) at 20×. Green represents ZO-1, red represents phalloidin, and blue represents DAPI. The scale bar represents 100 µm.

RNA-seq analyses showed that HBMEC treated with different levels of WSE for 24 h resulted in a significant decrease in gap junction markers, including *GJA4* and *GJA5*, and a significant decrease in tight junction markers, including *OCLN* and ZO-2/*TJP2*, while no significant changes were observed for *ZO-1/TJP1* (Figure 7A and Supplementary Figure S6) [60,61]. When examining hCMEC/D3 treated with WSE, a significant decrease in tight junction markers, including *ZO-2/TJP2* and *PECAM-1*, and a slight decrease in ZO*-1/TJP1* (*p*-value: 0.056) and the gap junction markers *GJA4* and *GJA5* were observed (Figure 7A and Supplementary Figure S6). Studies have shown that lithium chloride (LiCl) treatment can stabilize ZO-1 while the Gram-negative bacteria endotoxin, lipopolysaccharide (LPS), can destabilize ZO-1 in endothelial cells [62,63]. Thus, LiCl and LPS were used as positive and negative controls to stabilize and destabilize the ZO-1 in both HBMEC and hCMEC/D3 accordingly. While immunocytochemistry (ICC) images showed that there was a decreasing trend in ZO-1 expression with WSE treatment for 24 h in HBMEC, no significant changes were found through analysis on ImageJ (Figure 7B). No significant changes in ZO-1 expression level were observed through ICC in hCMEC/D3 treated with WSE for 24 h (Figure 7C).

## 3. Discussion

The goal of this study was to determine the acute effect of WSE in both HBMEC and hCMEC/D3. Based on previous studies on the effect of different PM sources in the brain and in brain endothelial cells, we hypothesized that WSE can induce proinflammatory cytokine production and disturb tight junctions in brain endothelial cells. From this study, we found that both HBMEC and hCMEC/D3 treated with WSE for 24 h resulted in a dose-dependent increase in IL-8 production and genes related to response to toxic substances, AhR pathway genes, and NRF2 response genes, but dose-dependent changes were much more prominent in HBMEC compared to hCMEC/D3. In contrast to upregulated genes, we found that downregulated changes were significantly different between HBMEC and hCMEC/D3. HBMEC showed a downregulation of genes that were related to the cell cycle while hCMEC/D3 showed a downregulation of immune response-related genes. Moreover, a comparison of untreated controls between HBMEC and hCMEC/D3 from RNA-seq analyses showed that HBMEC exhibited a higher number of tight junction and endothelial cell markers compared to hCMEC/D3. Although we observed the induction of proinflammatory cytokine production, the disturbance of tight junctions was not significant in both HBMEC and hCMEC/D3 at the protein level. RNA-seq analyses showed a reduction in tight junction genes (ZO-2/*TJP2* and *OCLN* in HBMEC and *ZO-2/TJP2* and *PECAM-1* in hCMEC/D3) and gap junction genes (*GJP4* and *GJP5* in HBMEC). Thus, our findings suggest that both HBMEC and hCMEC/D3 exhibit similar cellular inflammatory responses to WSE treatment for 24 h, but HBMEC could be a better in vitro model to study the effect of WSE in the brain endothelial cells compared to hCMEC/D3.

Out of 105 cytokines, IL-8 was the only proinflammatory cytokine that was significantly increased in HBMEC treated with WSE for 24 h when using the Proteome Profiler Human XL Cytokine Array Kit. IL-8 is a proinflammatory cytokine and is known to mediate inflammatory responses during infection, acute or chronic inflammation, and cancer [64,65,66]. An increase in IL-8 cytokine production was observed in both HBMEC and hCMEC/D3 for all doses of WSE treatment for 24 h, but dose-dependency and fold change were more significant in HBMEC compared to hCMEC/D3. Studies have shown that wood smoke and wildfire smoke exposure results in an increase in IL-8 in the serum, lung, and heart across different cells in vitro, animal models, and humans [67,68,69,70,71]. A recent study showed that human monocytic cells treated with urban wildfire ash resulted in a significant increase in IL*-8* and *CYP1A1* mRNA expression levels and AhR activity, which was also observed in our study, with an increase in CYP1A1 and other AhR pathway genes, including *AHRR*, seen in RNA-seq analyses for both HBMEC and hCMEC/D3 treated with WSE for 24 h [72]. Studies have shown that the activation of AhR can lead to increased IL-8 production [73]. Ambient PM_2.5_ resulted in an increase in IL-8 and *CYP1A1* and the activation of AhR in vitro and in vivo [74]. Airway epithelial cells exposed to wood smoke particles for 30 min resulted in an increase in the canonical targets of AhR, including CYP1A1 and *AHRR* [75]. AhR is known as a sensor for xenobiotics and is known to regulate and metabolize xenobiotics or toxicants [76]. AhR was initially discovered as a main receptor of an environmental toxicant, 2,3,7,8-tetrachlorodibenzo-*p*-dioxin, which is a type of halogenated aromatic hydrocarbon and known human carcinogen [76]. Thus, the activation of AhR through a canonical pathway initiates xenobiotic metabolism, leading to the synthesis of xenobiotic metabolism enzymes such as family 1-P450 cytochromes, cell proliferation, adhesion, cell migration, and the inhibition of the immune response [77].

Polycyclic aromatic hydrocarbons (PAHs), which are one of the major chemical components in wildfire smoke or wood smoke, including the smoldering eucalyptus wood smoke extract we used in our study, can bind to AhR [46,72,78,79]. Although many studies have investigated the role of AhR in the context of smoke-induced lung diseases, only a few studies have reported the role of AhR in the brain exposed to particulate matter, including wildfire smoke or wood smoke [7,80]. Urban particulate matter containing PAHs, AhR agonists, has been shown to regulate astrocytic activation and function in vitro [81]. Moreover, animal studies have shown that the intranasal instillation of nanoparticles decreased olfactory function with increased AhR expression level [82]. This same group reported that the intracranial administration of PM_2.5_ increased AhR expression and oxidative stress response in the temporal cortex of the brain [83]. In our work, we also noted that both HBMEC and hCMEC/D3 treated with wood smoke resulted in an increase in cellular response to oxidative stress accompanied by an increase in genes involved in the AhR and NRF2 pathways, and ferroptosis from RNA-seq analyses. Many of these upregulated genes are related to xenobiotic metabolism, which could suggest that cells are actively metabolizing wood smoke extracts but are also known to be involved in detecting the imbalance in ROS and antioxidants [84,85]. The NRF2 plays a significant role in regulating antioxidant enzymes and neutralizing ROS production [86]. Furthermore, ferroptosis is a programmed cell death induced by an imbalance between ROS and antioxidants and characterized by iron dependency and lipid peroxidation [87,88]. However, our DCFDA assay showed no significant changes in ROS production in HBMEC and hCMEC/D3 treated with WSE for 24 h. Thus, future studies examining antioxidant activity level, lipid peroxidation level, or time course ROS measurement could help to identify and understand the potential mechanisms of wood smoke extract in the brain endothelial cells.

The changes observed in downregulated genes were significantly different between HBMEC and hCMEC/D3 treated with WSE for 24 h. HBMEC resulted in a decrease in genes related to DNA replication and cell cycle checkpoints, while hCMEC/D3 resulted in a decrease in genes related to the humoral immune response and regulation of the innate immune response. Many of the cell cycle markers were significantly downregulated in HBMEC dose-dependently and consistently, but changes were not consistent and significant in hCMEC/D3. Additionally, biological process and pathway analysis showed an increase in prostaglandin metabolic process, prostaglandin synthesis and regulation, and prostaglandin and leukotriene metabolism in senescence in HBMEC. Conversely, pathway analysis showed that omega 6 fatty acid in senescence and prostaglandin and leukotriene metabolism in senescence were downregulated in hCMEC/D3. The upregulation of prostaglandin has been implicated in cell cycle arrest, including senescence [89,90]. Cellular senescence is characterized by stable growth arrest or cell cycle arrest in response to various intrinsic and extrinsic stimuli [91]. Some of the markers related to cell cycle arrest, including *CDKN2B* and *CDKN1A*, were upregulated, while *DHFR*, E2F2, and *E2F1* were downregulated in HBMEC. CDKN2B and CDKN1A are well-known genes that play a significant role in the cell cycle [92,93,94]. E2F transcription factor is known to regulate cell cycle progression and proliferation, and the dysregulation of E2F2 has been associated with endothelial cell senescence [56,95]. Decreased expression of DHFR and MTHFR is also indicated in endothelial cell senescence [57]. Studies have shown that endothelial cell senescence has been associated with aging and BBB alteration in vitro [96,97]. Moreover, cellular senescence has been implicated in the onset or aggravation of neurodegenerative disorders, including Alzheimer’s disease and Parkinson’s disease [98]. Thus, more studies need to be carried out to elucidate the potential role of cellular senescence induced by wood smoke or wildfire smoke in the brain endothelial cells.

Wildfire smoke exposure has been associated with an increased risk of dementia and cerebrovascular diseases and cognitive deficit within a few hours of exposure [9,11]. There is increasing evidence suggesting that air pollution is associated with many neurodegenerative diseases, including autism spectrum disorder, ADHD, Alzheimer’s disease, Parkinson’s disease, schizophrenia, and multiple sclerosis [99,100,101,102,103]. Although it is still unclear how these wildfire and wood smoke particles or chemicals deposited on the PM can cause neuroinflammation or deleterious effects in the brain, studies suggest that inhalation exposure to particulate matter can reach the brain through the olfactory system or travel systemically to disturb the BBB and enter the brain [87,104,105]. Our study did not find significant changes in the tight junction marker, ZO-1, from 24 h WSE treatment in both HBMEC and hCMEC/D3 via ICC at the protein level. Interestingly, ZO-1 was much more localized to the plasma membrane in HBMEC compared to the cytosolic expression of ZO-1 in hCMEC/D3 when visualized via ICC. Other studies also have shown that ZO-1 localization in the cytosol of hCMEC/D3 can depend on the conditions, and co-culture systems with astrocytes can help the localization of ZO-1 to the plasma membrane [106,107,108,109]. However, our RNA-seq analyses showed that HBMEC and hCMEC/D3 treated with WSE for 24 h resulted in a significant decrease in some of the tight junction markers (*OCLN* and *ZO-2* for HBMEC and *ZO-2* and *PECAM-1* for hCMEC/D3) at the transcript level. Moreover, hCMEC/D3 resulted in a decrease in ZO-1 (*p*-value: 0.056) but was not significant. A decrease in these tight junction markers could suggest increased BBB permeability [110,111,112]. A significant decrease in gap junction markers for *GJA4* and *GJA5* was observed in HBMEC, but not in hCMEC/D3. Gap junction proteins have been implicated in intercellular communication and the transduction of signals between the cells [30]. These findings could suggest that more significant changes in tight junctions could be induced if the cells were exposed to WSE for a longer period or higher dosage. Future studies should be conducted to determine the potential disturbance of the tight junctions in brain endothelial cells during longer exposure periods or higher dosages, as people are being exposed to more frequent and severe wildfire smoke.

Lastly, we compared the untreated controls between HBMEC and hCMEC/D3 for baseline transcript levels to better understand the differences observed for the two cell types we used in this study. Although studies have compared the primary HBMEC and hCMEC/D3 for their functional characteristics and uses for immune migration models or drug transport systems, no studies have compared the baseline transcript levels in the untreated control state using RNA-seq [113,114]. Both cell types expressed endothelial cell markers including tight junction markers, but HBMEC expressed much higher levels of endothelial cell markers and biological processes related to endothelial cells compared to hCMEC/D3 [115]. Similarly, another study has shown that hCMEC/D3 exhibited fewer tight junction markers compared to primary porcine brain capillary endothelial cells [116]. Furthermore, we observed that WSE treatment resulted in significant dose-dependency and fold changes in HBMEC compared to hCMEC/D3. The formation of a tight junction marker, ZO-1, around the plasma membrane was also better visualized in HBMEC compared to the cytosolic expression of ZO-1 in hCMEC/D3. Another study also has shown that primary brain endothelial cells express similar markers to 3D brain endothelial cell cultures which more closely represent the brain’s physiological state compared to 2D cultures and hCMEC/D3 [117]. Together, this could suggest that HBMEC could be a better in vitro model system for exploring and understanding the effect of WSE in brain endothelial cells. However, hCMEC/D3, which requires less maintenance and is a cost-effective model system, still resulted in similar molecular changes toward WSE treatment at both protein and transcript levels and could be utilized as an alternative to primary HBMEC.

## 4. Materials and Methods

### 4.1. Cell Culture

HBMEC were purchased from Cell Systems (Kirkland, WA, USA). HBMEC were plated in 96-well plates that were coated with an Attachment Factor™ (4Z0-201/4Z0-210) (Cell Systems, WA, USA). HBMEC were cultured in EGM™-2 Endothelial Cell Growth Medium-2 BulletKit™ (Lonza, Morristown, NJ, USA). hCMEC/D3 was purchased from Millipore Sigma (Burlington, MA, USA). hCMEC/D3 was cultured in a T75 flask after coating with rat collagen type I (Millipore Sigma, MA, USA) in PBS as directed up to P8. The culture medium for hCMEC/D3 consisted of EndoGRO™-MV Complete Media Kit (Millipore Sigma, MA, USA) supplemented with 1 ng/mL FGF-2 (Millipore Sigma, MA, USA). Both cultures were cultured at 37 °C in a humidified incubator with 5% carbon dioxide.

### 4.2. Smoldering Eucalyptus Wood Smoke Extract

Smoldering eucalyptus wood smoke condensates were generously provided by the US EPA. The wood smoke extract was generated by burning eucalyptus and collected during the smoldering phase as previously described by Kim et al. [46]. Chemical composition and particle analysis was performed and showed an average of 56–60% organic carbon content, which included levoglucosan and methoxyphenols along with semi-volatile compounds such as PAHs and N-Alkanes, and PM size was in the range of 1.02 µm [46]. Wood smoke extract condensate was collected in acetone. The solvent exchange was performed under a nitrogen source and resuspended in phosphate-buffered saline (PBS, ThermoFisher, Waltham, MA, USA) at 2 mg/mL. WSE was then aliquoted and kept frozen at −20 °C before use.

### 4.3. Experimental Design

Both HBMEC and hCMEC/D3 were plated in 96-well plates at 5 × 10^3^ cells/well. Cells were cultured in coated 96-well plates to confluency (3–5 days after seeding) before the treatment with eucalyptus WSE (10, 30, 50 µg/mL or 20, 70, 100 µg/mL), LPS (1 µg/mL), or LiCl (10 mM) that were resuspended in appropriate media for 24 h. LiCl and LPS were used as a positive and negative control to stabilize and destabilize ZO-1 accordingly in both HBMEC and hCMEC/D3. Controls were treated with an equal volume of appropriate media for each cell type. Doses were chosen based on previous studies conducted on brain or lung endothelial cells exposed to different sources of PM that resulted in cellular and molecular changes [35,36,118]. At the end of treatment, cells were fixed with 4% paraformaldehyde (PFA) for ICC, lysed to collect mRNA for bulk RNA sequencing, or supernatants were collected for ELISA and LDH assays. All experiments were repeated with N = 2–3 different seedings with n = 4–6 per treatment group.

### 4.4. Proteome Profiler Arrays

To assess cytokine production, a cytokine array experiment using a Proteome Profiler Human XL Cytokine Array Kit (R&D Systems, Minneapolis, MN, USA) was carried out following the manufacturer’s protocol with the collected cell supernatants. At the end of the experiment, arrays were imaged using a LI-COR Odyssey FC imager (Lincoln, NE, USA).

### 4.5. Cytokine Quantification

Cell supernatants were collected at the end of the experiment to measure the levels of secreted IL-8 using DuoSet ELISA kits (R&D Systems, MN, USA). Cytokine concentrations were derived from the absorbance values measured at 450 nm with a correction at 540 nm using an Agilent BioTek Synergy H1 microplate reader (Santa Clara, CA, USA) and converted to concentration values based on the standards provided by each kit.

### 4.6. Cytotoxicity Assay

Cells were cultured and treated as described above and 50 µL of cultured medium was collected at the end of the study for a cytotoxicity assay. Cells treated with lysis buffer from the CyQuant LDH Cytotoxicity Assay Kit (ThermoFisher, MA, USA) at 37 °C for 30 min served as maximum LDH activity controls. The collected supernatants including the maximum LDH activity controls were mixed with the 50 µL reaction mixture provided in the kit and left at room temperature for 30 min in the dark. After 30 min, the 50 µL stop solution provided in the kit was added. Absorbance values were measured at 450 nm using an Agilent BioTek Synergy H1 microplate reader (CA, USA).

### 4.7. DCFDA Assay

A DCFDA-H2DCFDA Cellular ROS Assay Kit (Abcam, Waltham, MA, USA) was used to measure ROS production in the cells treated with WSE for 24 h following the manufacturer’s protocol and measured using an Agilent BioTek Synergy H1 microplate reader (CA, USA) with an excitation/emission of 485/535 nm.

### 4.8. Immunocytochemistry

At the end of the experiments, the cell cultures were washed with Dulbecco’s phosphate-buffered saline (DPBS) and fixed with 4% PFA (20 min). Fixed cells were washed with 0.05% Tween-20 (Sigma-Aldrich, Saint Louis, MO, USA) solution in DPBS (wash buffer) (3×, 5 min), followed by a 3 min permeabilization with 0.1% Triton X-100 (ThermoFisher, MA, USA) solution in DPBS. Three additional washes were performed after the permeabilization step (5 min). Samples were blocked with 5% goat serum (ThermoFisher, MA, USA) in DPBS (blocking buffer) for 1 h. Samples were then incubated for an hour in a primary antibody solution containing mouse anti-ZO-1 (ThermoFisher, MA, USA) in blocking buffer (1:100). At the end of primary antibody incubation, samples were washed three times with wash buffer (5 min). Samples were then incubated with a secondary antibody solution containing goat anti-mouse antibodies conjugated to AlexaFluor 488 (ThermoFisher, MA, USA) in DPBS for an hour (1:500) followed by Phalloidin-647 (ThermoFisher, MA, USA) for 20 min in blocking solution (1:40). Samples were then washed three times with DPBS (5 min). Lastly, samples were incubated for 10 min with a drop of 4′,6-diamidino-2-phenylindole (DAPI) solution (ThermoFisher, MA, USA) to stain cell nuclei and washed with the wash buffer three times. All images were acquired with a Leica dmi6000b inverted fluorescence microscope at 20× magnification and analyzed using ImageJ (version 1.54f).

### 4.9. Bulk RNA-Sequencing, Data Processing, and Analyses

Total RNA was extracted from cells lysed in Qiagen Buffer RLT containing β-mercaptoethanol followed by RNA isolation using RNAeasy mini spin columns (Qiagen, Germantown, MD, USA). Two replicates per treatment group from three independent experiments were submitted for bulk RNA sequencing. Sequencing libraries were prepared using an Illumina Stranded mRNA prep, Ligation kit (Illumina, San Diego, CA, USA) and then sequenced on a NextSeq 500 (Illumina, CA, USA) with P3 reagent kits. Sequencing data quality was checked using FastQC software (version 0.12.0). The data were aligned to the human genome (hg38) using STAR (version 2.6.0c) [119] and mapped reads were counted using featureCounts [120]. Subsequently, the data were normalized using TMM normalization [121] and dosage-based clusters and outliers were identified by conducting a principal component analysis (PCA) using DESeq2’s (version 1.44.0) [122] function ‘PCAplot’ on both hCMEC/D3 and HBMEC. Based on the PCA, three outliers (two from the WSE 30 µg/mL and one from the WSE 50 µg/mL samples) for HBMEC were excluded from further analysis (Supplementary Figure S7). Differentially expressed genes were identified using DESeq2 [122]. A gene was considered as significantly differentially expressed when its false discovery rate adjusted *p*-value (FDR) was <0.05 and fold change was >1.5. A lower p.adjust value (typically < 0.05) indicates significant enrichment. Heatmaps were generated using the ‘pheatmap’ package (version 1.0.12) [123] in R (version 4.3.1) [124]. ClusterProfiler (version 4.12.6) [125], an R package, was used to perform gene set enrichment analysis and identify up- and downregulated biological processes, cellular components, and pathways. Enrichment analysis was conducted separately for each gene list using the ‘enrichGO’ function to assess GO terms and the ‘enrichWP’ function to assess WikiPathways terms. Significantly enriched terms were identified based on an adjusted *p*-value threshold of 0.05. Dot plots visualizing the enriched biological processes, cellular components, and pathways were generated using the ggplot2 package (version 3.4.4) [126] in R. The ggplot2 function ‘geom_point’ was utilized to create the dot plots, with each dot representing a specific GO term or pathway and the size of the dot corresponding to the number of genes associated with the term. The dot plots were created using a color scale ranging from red to blue, where red represented the most significantly enriched terms and blue represented the least significantly enriched terms. The count represents the number of genes from our input list that were found in a specific gene set or pathway. A higher count suggests a stronger association with biological relevance.

### 4.10. Statistical Analysis

The differences between treatment groups were evaluated by one-way ANOVA with post-Tukey’s test (GraphPad Software, Inc., La Jolla, CA, USA, version 10.0.0). Statistical tests are specified in the figure legends.

## 5. Conclusions

To our knowledge, this was the first study to investigate the acute effect of WSE in brain endothelial cells using HBMEC and hCMEC/D3. Our study found that 24 h WSE treatment induced IL-8 production and increased genes involved in the AhR and NRF2 pathways in both HBMEC and hCMEC/D3 dose-dependently, but the effects were more significant in HBMEC compared to hCMEC/D3. Interestingly, some of the genes related to cell cycle and senescence were significantly downregulated in HBMEC after 24 h WSE treatment, which was not observed in hCMEC/D3. The effect of WSE in the tight junctions of HBMEC and hCMEC/D3 was unclear as we did not see significant changes in ZO-1 at the protein level, but significant changes were observed at the transcript level for tight junction markers in both HBMEC (*OCLN* and *ZO-2*) and hCMEC/D3 (*PECAM-1* and *ZO-2*). Although many genes related to ROS imbalance were increased at the transcript level, no changes were observed in ROS production from 24 h WSE treatment on both cell types via the DCFDA assay. Thus, future work is needed to elucidate the specific molecular mechanisms of proinflammatory cytokine production and to determine the potential disturbance of tight junctions with longer exposure to WSE treatment in HBMEC and hCMEC/D3. From this study, we found that both HBMEC and hCMEC/D3 exhibited similar responses to WSE, and both can be utilized to explore and understand the potential delivery route of particulate matter, including wildfire smoke and wood smoke particles, to the brain, and to study the molecular mechanism of neuroinflammation from wildfire smoke or wood smoke exposure. Moreover, a comparison of the untreated controls between HBMEC and hCMEC/D3 via RNA-seq analyses showed increased tight junction and endothelial cell function markers in HBMEC, suggesting that the use of primary HBMEC could be a better in vitro model system to study the effect of WSE on brain endothelial cells compared to hCMEC/D3. While our findings showed that WSE elicits dose-dependent effects in brain endothelial cells, only one brain cell type was used in this study. Other brain cell types should also be looked at together to understand potential neurotoxicity and crosstalk between the cells. Furthermore, wildfire smoke PM_2.5_ significantly varies in composition depending on the location, weather, fuel, and temperature of the fire [127]. Thus, different types of biomasses other than smoldering eucalyptus should be assessed to identify which biomass can be more toxic to the brain. Lastly, future studies should incorporate chronic time points to determine the potential chronic effects of wildfire smoke on the brain.

## Data Availability

The datasets presented in this study can be found in online repositories. The names of the repository/repositories and accession number(s) can be found below: NCBI Gene Expression Omnibus (GEO), GSE271935.

## Supplementary Materials

The following supporting information can be downloaded at: https://www.mdpi.com/article/10.3390/ijms251910288/s1.
