# Supplementary material for: Eucalyptus Wood Smoke Extract Elicits a Dose-Dependent Effect in Brain Endothelial Cells
Source: Int J Mol Sci. 2024 Sep 24;25(19):10288. doi: 10.3390/ijms251910288 (PMC11476751; doi:10.3390/ijms251910288)
Supplement: Supplementary file 1 [file ijms-25-10288-s001.zip › ijms-3177257-supplementary.pdf]

Supplementary Materials

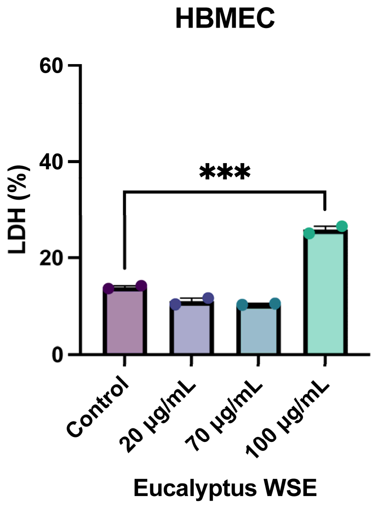

Figure S1. HBMEC treated with 20, 70, 100 μg/mL of WSE for 24h. LDH activity measured from cell supernatants in HBMEC (n=2, ***$p<0.001$ compared to controls using one-way ANOVA with post-Tukey's test).

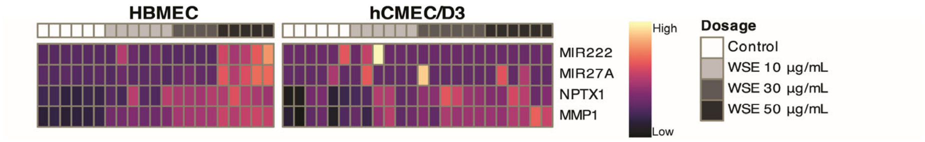

Figure S2. Differentially expressed genes in HBMEC and hCMEC/D3 treated with different levels of WSE (10, 30, 50 μg/mL) for 24h. A) Heatmaps of genes that related to brain injury or particulate exposure in the brain.

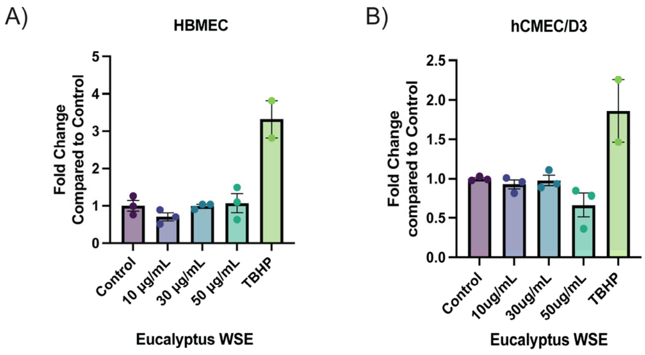

Figure S3. ROS measured using the DCFDA assay in A) HBMEC and B) hCMEC/D3 treated with different levels of WSE (10, 30, 50 μg/mL) for 24h (n=3). Tert-Butyl Hydrogen Peroxide was used as a positive control.

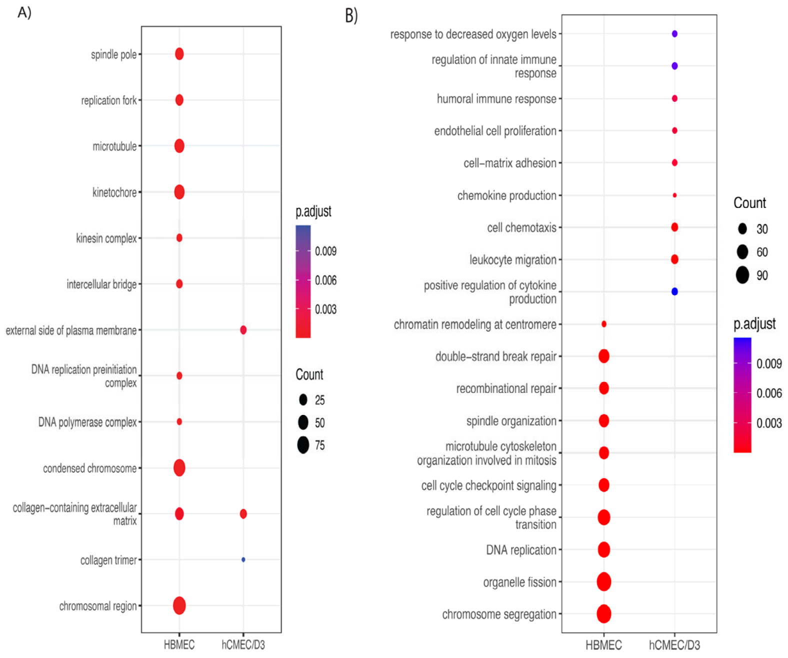

Figure S4. RNA-seq analyses of downregulated DEGs. A) Significant and relevant terms in cellular component between HBMEC and hCMEC/D3. B) Significant and relevant terms in biological process between HBMEC and hCMEC/D3.

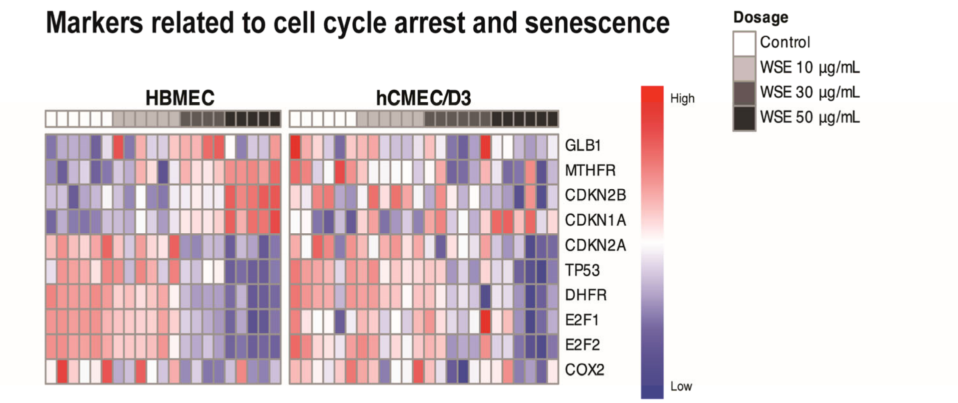

Figure S5. Differentially expressed genes in HBMEC and hCMEC/D3 treated with different levels of WSE (10, 30, 50 µg/mL) for 24h. A) Heatmap of genes that are related to cell cycle arrest and senescence.

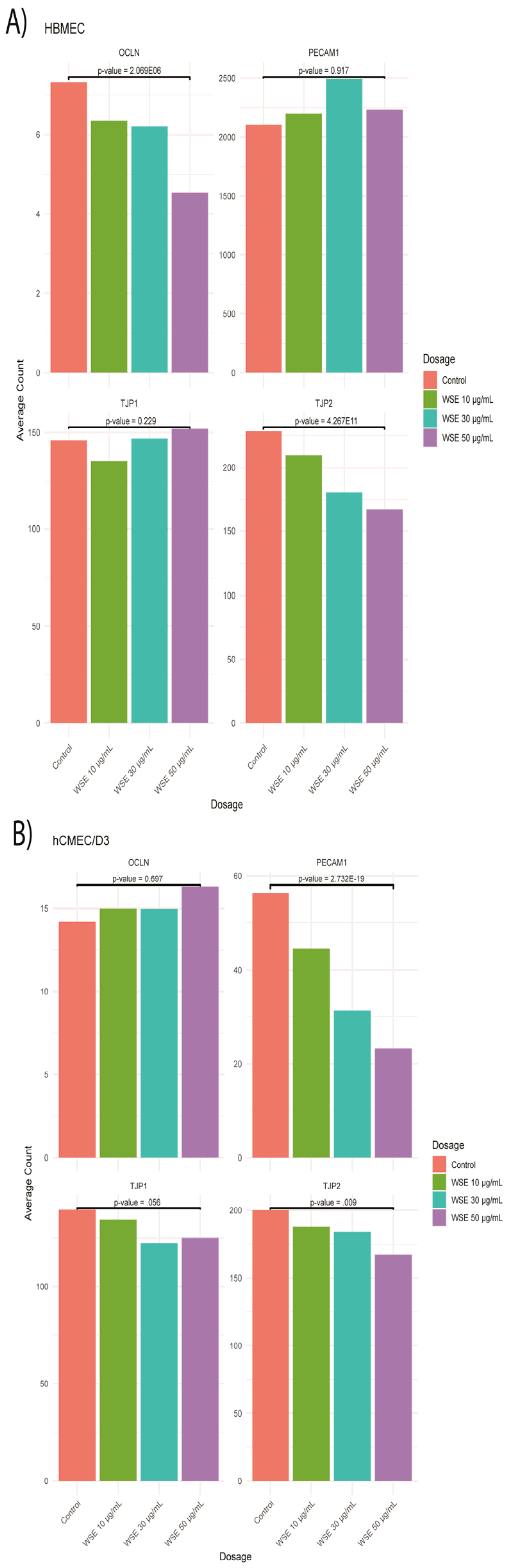

Figure S6. Average count of endothelial cell and tight junction markers in HBMEC and hCMEC/D3 treated with WSE for 24h.

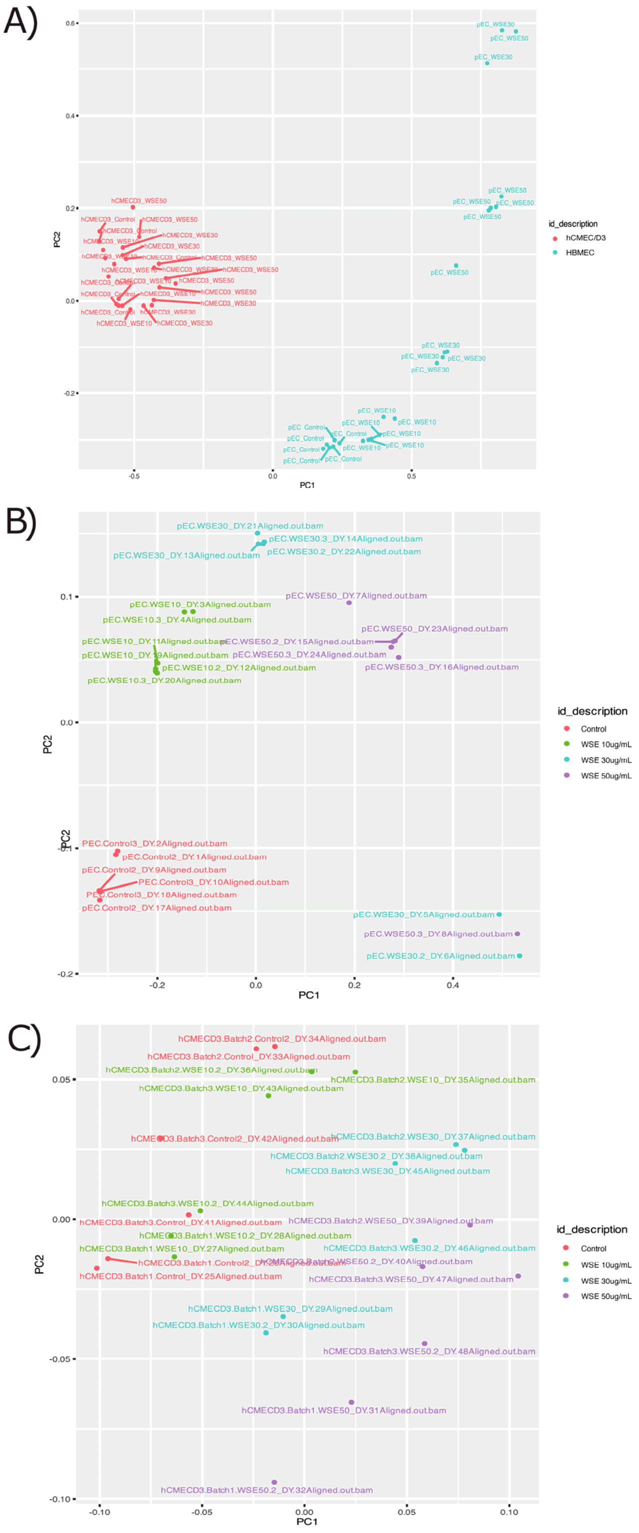

Figure S7. PCA plot of WSE treated (10, 30, 50 μg/mL) cells A) HBMEC and hCMEC/D3 together B) HBMEC only and C) hCMEC/D3 only.
